# Voices for Health Equality

**Published:** 2006-06-15

**Authors:** Bronx Health REACH

## About the Video

Bronx Health REACH is a coalition of 40 community- and faith-based organizations founded in 1999 that is focused on eliminating racial and ethnic health disparities in the southwest Bronx.  As part of its community education effort, the coalition produced a video, titled *Voices for Health Equality,* which depicts the reality of health disparities from the perspective of both community members and healthcare professionals. The video is an effective tool for sharing the coalition's goals and introducing the problem of racial disparities to community leaders and policy makers.

**Figure:**
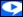
Watch* Voices for Health Equality* (RM 14Mb) You will need RealOne Player to view these videos. Learn more about RealPlayer. Learn more about RealPlayer.

## Transcript

**Mr. James Warren, Patient, Walton Family Health Center**

Doctor came out. She told him what happened; I told him. He asked me who my doctor was and went back in the door and left me standing there. Nobody said anything. You know, it was just, you know…

**Ms. Jeanette Puryear, Executive Director, Mid-Bronx Senior Citizens Council**

One of the things it seems to me you learn as a black professional is that as long as you are in your professional clothing, or you're in a particular place, you may be treated in a particular way. However, when you go to the emergency room or when you are in contact with certain kinds of elements of our society and you are regarded just as a person of color, the treatment is very different. So what happened at this hospital was that once I got there and took off my professional clothing and I was like everybody else, I stayed there for hours—this is one of the best hospitals here in the Bronx. I was in the emergency room for 24 hours.

**Ms. Joyce Davis, President, Joyce Davis Associates**

You know, we did some focus groups when we first started this project. And if you could hear the stories of the people who sat in those groups, when you ask them certain questions about how do they feel, you know, when they go to a doctor, what kind of treatment do they get, what would they like to see different, what's going on. Most people, originally—some of them, many of them—didn't even understand that they were not getting the kind of treatment that they are supposed to get.

**Ms. Evelyn Laureano, Executive Director, Neighborhood SHOPP**

The consequence of not having the information for the clients that we see at Neighborhood SHOPP, primarily Hispanic and African American elders, is death at an earlier age or increased functional incapacity as a result of the impact of the illness over the course of their life.

**Ms. Magdelena Torres, Community Health Advocate, Highbridge Community Life Center**

During the years, I learned some good things and I also learned bad things, like they would give me wrong information at my doctor's office about the levels of sugar and, you know, that were right for me to have. And over time those caused damage. I have neuropathy. I have retinopathy. I nearly went blind, and now I suffer with debilitating pains in my feet from the nerve damage that occurred.

**Dr. Neil Calman, President & Co-Founder, Institute for Urban Family Health**

It's very common to go into a teaching hospital in New York and have Medicaid patients still referred to today as "teaching patients." You go on the ward and you say, "These are the teaching patients," and "the teaching patients" mean these are people with Medicaid or people who are uninsured. Now, they could be, you know, on the same floor or in the same room as somebody else, but they're called teaching patients because they're poor, or they're on Medicaid, or they're uninsured. And in New York, you're much more likely to be uninsured or on Medicaid if you're a person of color.

**(CDC Representative's name was not given)**

Bronx Health REACH is a project that was funded by the CDC in 1999 to look at the problems of racial and ethnic disparities in health outcome. We're located in the southwest Bronx, we cover a 4–ZIP-code target area that includes about 275,000 people, and we run a number of community-based health education and public health initiatives to educate people about health disparities.

## Author Information

For more information about the video, please contact Maxine Golub, MPH, Institute for Urban Family Health, Bronx Health REACH, 16 East 16th Street, New York, New York, 10003. Telephone: 212-633-0800, Ext. 286. E-mail: mgolub@institute2000.org.

